# Knockdown of glucose-regulated protein 78 abrogates chemoresistance of hypopharyngeal carcinoma cells to cisplatin induced by unfolded protein in response to severe hypoxia

**DOI:** 10.3892/ol.2013.1753

**Published:** 2013-12-11

**Authors:** LIHONG PI, XIAOMING LI, QI SONG, YUPENG SHEN, XIUYING LU, BIN DI

**Affiliations:** 1 Department of Otorhinolaryngology, Hebei Medical University, Shijiazhuang, Hebei 050017, P.R. China; 2 Department of Otolaryngology Head and Neck Surgery, Bethune International Peace Hospital, Shijiazhuang, Hebei 050082, P.R. China

**Keywords:** glucose-regulated protein 78, chemoresistance, hypopharyngeal neoplasms, hypoxia, unfolded protein response

## Abstract

Hypoxia renders tumor cells with reduced sensitivity and increased resistance to chemotherapeutic agents. One of the possible mechanisms underlying this unfavorable status is activation of the unfolded protein response (UPR) under hypoxic conditions, due to the upregulation of glucose-regulated protein 78 (GRP78) expression. GRP78, an endoplasmic reticulum chaperone protein and a key regulator of the UPR, has been reported to be overexpressed in various types of cancer. However, the role of GRP78 in regulating the cell growth and apoptosis of hypopharyngeal carcinoma cells, with regard to the severity of hypoxia, remains unclear. Therefore, the aim of the present study was to investigate whether, and under what circumstances, GRP78 is associated with hypoxia-induced chemoresistance in hypopharyngeal carcinoma. For this purpose, cells from the FaDu human hypopharyngeal carcinoma cell line were cultured under normoxic and hypoxic conditions for different time periods. No significant changes in GRP78 and C/EBP homology protein (CHOP) protein expression levels were revealed under moderately hypoxic conditions (oxygen concentration, 1%), but these levels were changed over time under severely hypoxic conditions (oxygen concentration, <0.02%). This indicated that severe hypoxia, rather than moderate hypoxia, leads to UPR activation in hypopharyngeal carcinoma cells. Knockdown of GRP78 with short hairpin RNA inhibited cell proliferation and promoted apoptosis under severely hypoxic conditions, even with cisplatin treatment, indicating that GRP78 confers FaDu cells resistant to chemotherapy in response to severe hypoxia. Furthermore, knockdown of GRP78 resulted in a significant increase in CHOP and Bax expression levels and a decrease in Bcl-2 expression levels with simultaneous increase in the levels of apoptosis under severely hypoxic conditions. It was concluded that severe hypoxia leads to UPR activation and elevation of GRP78 expression, promoting cell survival and inducing chemoresistance. Silencing of GRP78 may block the pro-survival arm of UPR, simultaneously promoting proapoptotic signaling through induction of CHOP. Downregulation of GRP78 may be a promising strategy for overcoming the resistance of hypopharyngeal cancer to chemotherapy.

## Introduction

Hypopharyngeal carcinoma is a type of malignancy that arises from the mucosal epithelia in the hypopharynx (1). At present, in addition to surgery, adjuvant chemotherapy is important for the multidisciplinary treatment of hypopharyngeal cancer. However, the resistance of cancer cells to chemotherapeutic agents remains an unquestionable entity, which hampers treatment planning and successful tumor control in routine clinical practice. Among various causes responsible for therapeutic resistance, hypoxia of the cancer microenvironment is proposed to be important (2). Hypoxia evokes a series of intracellular signal cascades, resulting in cell cycle arrest, limited diffusion of drugs and changes in gene transcription activity, which contributes to tumor growth, angiogenesis and metastasis and, thereby, enhances the resistance of cancer cells to chemotherapeutic drugs. Previous studies have revealed that tumor cells adapt to hypoxia by hypoxia-inducible factor (HIF)-dependent and -independent pathways (3,4). It has been previously demonstrated that HIF-1α is induced by mild to moderate hypoxia with oxygen concentrations ranging between 5 and 0.1%, *in vitro* and *in vivo*. By contrast, the induction of HIF-1α in response to severe hypoxia (oxygen concentration, <0.02%) is considered a functionally different state (3,5) and requires further investigation.

Due to an insufficient blood supply and poor oxygenation in solid tumors, including head and neck squamous cell carcinoma (HNSCC), severe hypoxia in the tumor region, particularly in the central section of the tumor bulk, is a persisting status. Although cancer cells are considered to be unable to survive in tumor tissues with extremely low oxygen concentrations, they are well adapted to complete anoxia by HIF-independent pathways, for example the unfolded protein response (UPR), and exhibit prolonged survival times (6). It has been previously identified that hypoxia activates UPR and causes an increase in glucose-regulated protein 78 (GRP78) expression, the latter of which is also referred to as the immunoglobulin heavy chain binding protein. GRP78 is an important molecular chaperone that resides in the endoplasmic reticulum (ER) and belongs to the HSP70 protein family. In non-stressed cells, GRP78 binds to three transmembrane ER sensors, PERK, IRE1a and ATF6, and maintains them in an inactive form. In tumor cells suffering from stresses, including glucose starvation, hypoxia and oxidative stimulation, unfolded proteins accumulate in the ER lumen, which, in turn, causes dissociation of GRP78 from the three ER transmembrane sensors, leading to their activation and triggering of UPR (7).

GRP78, the most important marker of UPR, has been reported to be elevated in a variety of tumors, including breast (8), ovarian (9) and prostate (10). In human cancers, GRP78 protects tumor cells from apoptosis, contributing to tumor cell proliferation, survival and therapeutic resistance. However, the roles of GRP78 in regulating the growth and survival of hypopharyngeal carcinoma cells with regard to the severity of hypoxia remain to be elucidated. The aim of the present study was to investigate the changes of GRP78 in response to various conditions of hypoxia, and its association with chemoresistance in hypopharyngeal carcinoma. It was demonstrated that the induction of GRP78 by severe hypoxia is a cause of chemoresistance and knockdown of GRP78 with short hairpin RNA (shRNA) enhances the chemosensitivity of hypopharyngeal carcinoma cells to cisplatin (DDP) in response to severe hypoxia.

## Materials and methods

### Cell line and lentivirus infection

FaDu hypopharyngeal squamous carcinoma cell line was purchased from the Type Culture Collection of the Chinese Academy of Sciences (Shanghai, China). Cells were grown at 37°C in a CO_2_ incubator with 5% CO_2_ and 95% humidity and Dulbecco’s modified Eagle’s medium (with 4.5 mg/ml glucose) supplemented with 10% fetal bovine serum (Hangzhou Sijiqing Biology Engineering Materials Co., Ltd., Hangzhou, China), 100 U/ml penicillin, 100 μg/ml streptomycin and 4 mM L-glutamine (Gibco, Paisley, United Kingdom). The shRNA eukaryotic plasmid expression vector kit was purchased from GeneCopoeia, Inc. (Guangzhou, China). For infection, 3×10^5^/2 ml FaDu cells were plated in six-well plates, infected with the lentivirus containing a shRNA targeting GRP78 (shGRP78; 5′-TTCCGGTCTACTATGAAGC-3′) or a scrambled oligonucleotide (scshRNA; 5′-GCTTCGCGCCGTAGTCTTA-3′) and sorted by flow cytometry to ensure 100% positivity. Established stable cell lines were named GRP78(+/+) and GRP78(−/−) and cultured in the medium described above (containing 2.5 μg/ml puromycin) for subsequent usage.

### Hypoxia incubation and DDP treatment

Hypoxic conditions were achieved and maintained by regulating the gas flow rates under 95% N_2_ and 5% CO_2_. The oxygen level within the incubation chamber was monitored with an anaerobic indicator (Zirconia Oxygen Analyzer; Nanjing Nensite Instrument Co., Ltd., Nanjing, China). To acquire moderate hypoxia, gas purging (3 l/min) with 95% N_2_ and 5% CO_2_ was performed for 8–9 min until the oxygen level reached 1%, when the purge gas was replaced by a gas mixture of 1% O_2_, 94% N_2_ and 5% CO_2_. The gas flow rate was adjusted steadily at 0.8 l/min and oxygen levels were maintained at 1%. To achieve severe hypoxia, gas purging (3 l/min) was performed for 12–13 min until the oxygen level reached 0.02% and, simultaneously, the gas flow rate was adjusted steadily at 0.8 l/min. The oxygen level had to be maintained for the duration of the procedure for each separate experiment.

To investigate the effect of various hypoxic conditions on the protein expression of GRP78 and C/EBP homology protein (CHOP), FaDu cells were cultured in six-well plates under moderate and severe hypoxia for the indicated time periods. For immunocytochemical analysis, cover glasses were placed in the wells of the six-well plates, to which 3.0×10^5^ GRP78(+/+) or GRP78(−/−) cells were seeded per well and grown overnight in an incubator (37°C and 20% O_2_), followed by culture under severely hypoxic conditions for 24 h. To investigate the effects of GRP78 on the chemosensitivity of hypopharyngeal carcinoma cells, GRP78(+/+) and GRP78(−/−) cells were seeded in 96-well plates at a density of 5×10^3^ per well, incubated under severe hypoxia and treated with various concentrations of DDP for 24 h. In the additional experiments, including cell apoptosis and protein regulation assays, GRP78(+/+) and GRP78(−/−) cells were plated at 3×10^5^ cells/well in six-well plates and treated with DDP (40 μmol/l) under severe hypoxia for 24 h.

### Fluorescent quantitative real-time polymerase chain reaction (qPCR)

Total RNA was isolated using TRIzol reagent (Invitrogen Life Technologies, Carlsbad, CA, USA) and the cDNA was synthesized by reverse transcription according to the manufacturer’s instructions for the HiFi-MMLV cDNA kit (cat. no. CW0744; CoWin Biotech Co., Ltd., Beijing, China). qPCR was performed using the ABI Prism 7500HT Sequence Detection system (Applied Biosystems, Inc., Foster City, CA, USA). Reactions were performed in 20 μl volumes. qPCR parameters were as follows: Initial denaturation at 95°C for 10 min, followed by 40 cycles of 95°C for 15 sec and 60°C for 60 sec. The mRNA expression level was determined using the 2^−ΔΔCt^ method and actin was used as internal control. The primers used for qPCR were as follows: Forward: 5′-CTGTAGCGTATGGTGCTGCTGTCC-3′ and reverse: 5′-TGACACCTCCCACAGTTTCAATACCA-3′ for GRP78; and forward: 5′-ACTTAGTTGCGTTACACCCTT-3′ and reverse: 5′-GTCACCTTCACCGTTCCA-3′ for actin.

### Western blot analysis

Cells from corresponding experiments were harvested and washed with ice-cold phosphate-buffered saline (PBS) and total protein was extracted with ice-cold lysis buffer. Protein concentrations were quantified according to the manufacturer’s instructions for the BCA protein assay kit (Pierce Biotechnology Inc., Rockford, IL, USA). For protein denaturation, the mixture of total protein and 3X loading buffer [0.1 M Tris-Cl, (pH 6.8), 4% SDS, 0.2% bromophenol blue and 20% glycerol] was heated at 97°C for 5 min in a Thermomixer comfort (Eppendorf, Hamburg, Germany). Equal amounts of protein (30 μg) were separated by 12% SDS-PAGE and transferred to a polyvinylidene fluoride membrane, the latter of which was blocked in 5% skimmed milk for 2 h at 37°C. Immunoblotting was performed using primary antibodies, including GRP78 (1:1000), CHOP/GADD153 (1:200) and HIF-1α (1:1000) (all Abcam, Cambridge, UK), and Bcl-2, Bax and β-actin (all 1:200; Santa Cruz Biotechnology, Inc., Santa Cruz, CA, USA). Horseradish peroxidase-conjugated goat anti-mouse or anti-rabbit antibody was used as a secondary antibody (1:5000; Beijing Zhongshan Goldenbridge Bio-technology Co., Ltd., Beijing, China). Protein expression levels were detected using the RPN2132 enhanced chemiluminescence plus western blotting detection system (GE Healthcare Life Sciences, Amersham, UK). The bands were exposed to film in a dark chamber and the relative expression levels of each separate protein were determined by densitometry using a gel image analyzing system (UVP, LLC, Upland, CA, USA).

### Immunocytochemistry

Cover glasses grown with GRP78(+/+) and GRP78(−/−) cells were washed twice with PBS (pH 7.4) and fixed with 4% paraformaldehyde for 20 min. Cells on cover glasses were permeabilized with 0.3% Triton X-100 for 30 min and incubated with goat serum for 20 min at 37°C to block the non-specific binding of the antibody. Subsequently, cells were incubated with primary antibody (rabbit antihuman GRP78 polyclonal antibody, 1:500; and mouse antihuman CHOP monoclonal antibody, 1:100; Abcam) at 4°C overnight. Following washing, cells were incubated with goat-anti-rabbit antibody for 10 min at room temperature, labeled with horseradish-peroxidase-labeled pronase avidin and 3,3′-Diaminobenzidine reagent. Cells were then counterstained with hematoxylin for 5 min and dehydrated with gradient alcohol solutions. Cover glasses were mounted onto the glass slide with neutral resin and visualized using an optical microscope (magnification, ×200; VANOX-S, Olympus, Tokyo, Japan). Optical density (OD) values were measured using Image-Pro Plus software (Media Cybernetics Inc., Rockville, MD, USA).

### MTT cell viability assay

MTT reagent (20 μl; 5 mg/ml; Sigma-Aldrich, St. Louis, MO, USA) was added to each well of a 96-well plate, 4 h prior to the indicated time points. Following 4 h of incubation at 37°C, the culture medium was removed from each well and the precipitate from each representative well was dissolved in 150 μl dimethyl sulfoxide. The OD values were measured at 409 nm using an enzyme-linked immunosorbent detector (Model 550; Bio-Rad, Hercules, CA, USA).

### Flow cytometry of cell apoptosis

Treated cells in each well were trypsinized and collected by centrifugation at 200 × g for 4 min. Next, the harvested cells were washed twice with cold PBS and fixed with 70% ethanol at 40°C overnight. Cells were resuspended in PBS containing 40 μg/ml propidium iodide (PI), 0.1 mg/ml RNase A and 0.1% triton X-100 in a dark room for 30 min at 37°C. Counting of apoptotic cells was carried out by measuring sub-G1 DNA content using the PI method with a FACS Aria flow cytometer (Becton-Dickinson, Franklin Lakes, NJ, USA).

### Statistical analysis

Data are presented as the mean ± standard deviation. One-way analysis of variance and Student’s t-test were performed for statistical evaluation of data. Statistical comparisons were performed using SPSS software for Windows (version 13.0; SPSS, Inc., Chicago, IL, USA), and P<0.05 was considered to indicate a statistically significant difference.

## Results

### Establishment of FaDu hypopharyngeal carcinoma cell line with knockdown of GRP78

**Figure 1:**
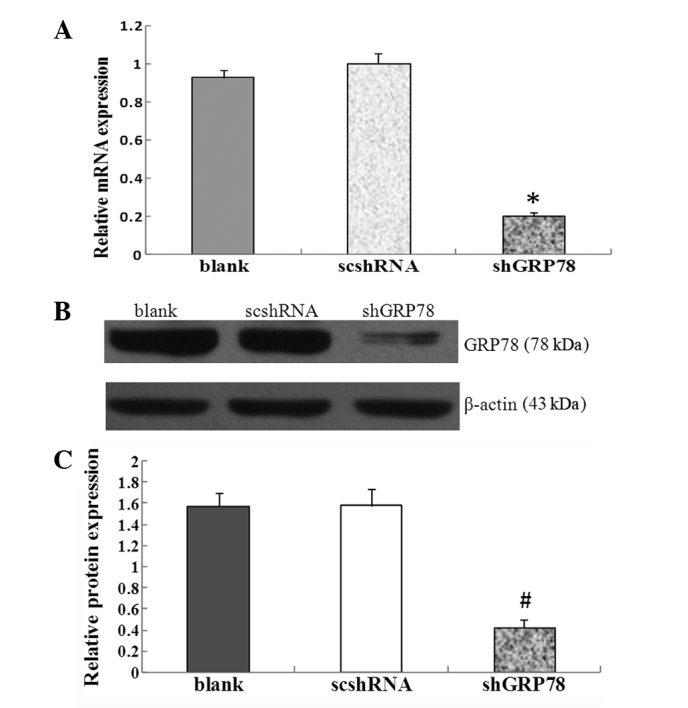
Generation of stable shRNA-expressing cell lines with or without knockdown of GRP78 in FaDu cells. FaDu cells were stably transfected with expression plasmids containing shRNA targeting GRP78 and its control scramble sequence. (A) qPCR determined the relative GRP78 mRNA expression levels of blank, scshRNA and shGRP78 FaDu cells. (B) Western blotting determined the relative GRP78 protein expression levels of blank, scshRNA and shGRP78 FaDu cells. (C) Histogram of the relative protein expression levels of GRP78 by western blot analysis in blank, scshRNA and shGRP78 groups. Data shown are from three individual experiments. ^*^P<0.01, vs. scshRNA; ^#^P<0.01, vs. scshRNA. GRP78, glucose-regulated protein 78; shRNA, short hairpin RNA; scshRNA, scrambled shRNA; qPCR, quantitative real-time polymerase chain reaction.

To investigate the role of GRP78 in regulating the proliferation and growth of FaDu cells under hypoxic conditions, lentivirus containing shGRP78 or scshRNA was used to infect FaDu cells, generating stable cell lines defined as GRP78(−/−) and GRP78(+/+). As demonstrated by qPCR and western blot analysis, downregulation of GRP78 was at mRNA and protein levels in GRP78(−/−) cells as opposed to GRP78(+/+) cells. In quantitative analysis, the GRP78 shRNA plasmid caused a significant reduction in GRP78 expression at mRNA (P<0.01) and protein (P<0.01) levels under normoxic conditions (Figs. 1A and B). It was demonstrated that the hypopharyngeal carcinoma cell line with knockdown of GRP78 [GRP78(−/−)] was successfully established.

### Effects of various hypoxic conditions on the expression of major proteins in UPR pathways in hypopharyngeal carcinoma cells

**Figure 2:**
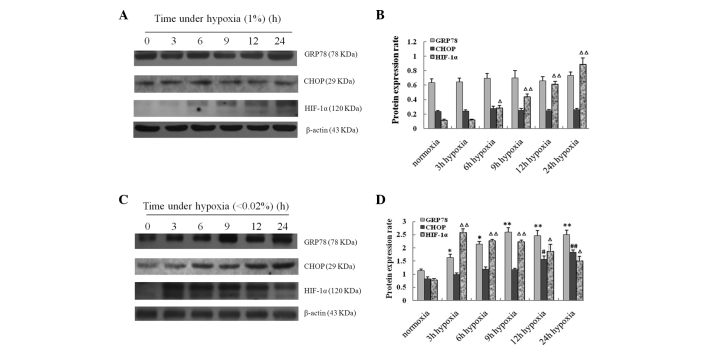
Changes of GRP78, CHOP and HIF-1α expression levels in FaDu cells following exposure to moderate and severe hypoxia for 3, 6, 9, 12 and 24 h, respectively. Expression of GRP78, CHOP and HIF-1α were detected by western blot analysis. Results from western blot analysis in FaDu cells exposed to (A) moderate and (C) severe hypoxia. (B and D) Histograms of the expression levels of GRP78, CHOP and HIF-1α proteins in FaDu cells determined by western blot analysis. Data shown are from three individual experiments. ^*^P<0.05 and ^**^P<0.01, vs. normoxic conditions (GRP78); ^#^P<0.05, ^##^P<0.01, vs. normoxic conditions (CHOP); ^Δ^P<0.05, ^ΔΔ^P<0.01, vs. normoxic conditions (HIF-1α). GRP78, glucose-regulated protein 78; CHOP, C/EBP homology protein; HIF-1α, hypoxia-inducible factor-1α.

GRP78 and CHOP are two main regulator proteins downstream of the UPR pathways. The expression of GRP78 and CHOP under various hypoxic conditions was investigated in FaDu cells by western blot analysis. Under normoxic conditions, the expression of GRP78 and CHOP remained almost at the same level for up to 24 h (Fig. 2A). However, expression of GRP78 and CHOP was upregulated in FaDu cells under severe hypoxia (Fig. 2C). The expression of GRP78 protein, a marker in pro-survival signaling of UPR, increased gradually under severe hypoxia between 3 and 9 h and remained almost unchanged for up to 24 h. Severe hypoxia also leads to the induction of CHOP, a major proapoptotic arm of the UPR. CHOP protein levels were observed to increase gradually between 12 and 24 h, and peaked at 24 h (Fig. 2C). Under moderate hypoxia, HIF-1α, an important marker of hypoxia, increased gradually between 6 and 24 h, and peaked at 24 h. However, under severe hypoxia, the expression of HIF-1α was markedly increased within 3 h, maintained at an even level between 6 and 12 h, and significantly reduced at 24 h. This indicated that in the later stages of severe hypoxia, HIF-1α-independent pathways, including UPR, may dominate the response of the cell to hypoxic conditions.

### Induction of GRP78 by severe hypoxia is associated with chemoresistance of hypopharyngeal carcinoma cells to DDP

**Figure 3:**
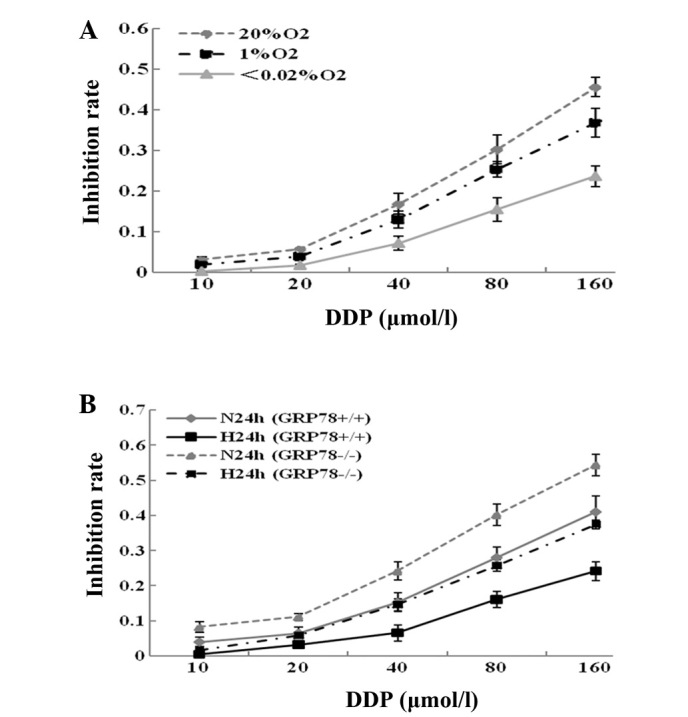
Results of cell proliferation inhibition assays by MTT. (A) FaDu cells were treated with 10, 20, 40, 80 and 160 μmol/l of DDP for 24 h under normoxia, and moderate and severe hypoxia. Compared with FaDu cells treated under normoxic conditions, the proliferation inhibition rates of FaDu cells were significantly decreased under moderate (P<0.05) and severe (P<0.01) hypoxia. (B) GRP78(+/+) and GRP78(−/−) hypopharyngeal carcinoma cells were treated with various concentrations (10, 20, 40, 80 and 160 μmol/l) of DDP for 24 h under normoxia and severe hypoxia. Results showed that knockdown of GRP78 with shRNA greatly increased cell proliferation inhibition induced by DDP under normoxia and severe hypoxia in hypopharyngeal carcinoma cells (all P<0.01). All experiments were performed in triplicate. GRP78, glucose-regulated protein 78; N24h, normoxia for 24 h; H24h, severe hypoxia for 24 h; shRNA, short hairpin RNA; DDP, cisplatin.

**Figure 4:**
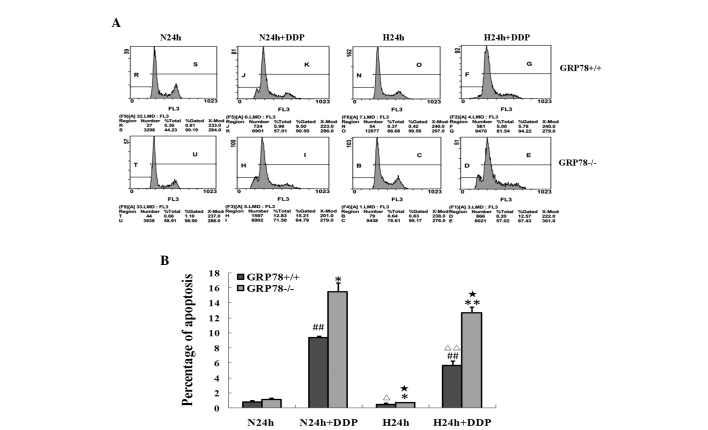
Knockdown of GRP78 sensitizes hypopharyngeal carcinoma cells to DDP-induced apoptosis. GRP78(+/+) and GRP78(−/−) FaDu cells were treated with DDP (40 μmol/l) for 24 h under normoxic or severely hypoxic conditions, and apoptosis was measured by the propidium iodide method using flow cytometry. (A) Apoptosis of GRP78(+/+) and GRP78(−/−) cells was determined by flow cytometry. (B) Histogram of the apoptosis rates in the GRP78(+/+) and GRP78(−/−) cells determined by the results from A. ^*^P<0.05 and ^**^P<0.01, vs. GRP78(+/+); ^##^P<0.01, N24h + DDP vs. N24h or H24h + DDP vs. H24h in GRP78(++); ^Δ^P<0.05 and ^ΔΔ^P<0.01, H24h vs. N24h or H24h + DDP vs. N24 + DDP in GRP78(+/+) cells; ^«^P<0.05, N24h vs. H24h or N24h+DDP vs. H24h+DDP in GRP78(−/−) cells. Data shown are from three individual experiments. GRP78, glucose-regulated protein 78; DDP, cisplatin; N24h, normoxia for 24 h; H24h, severe hypoxia for 24 h.

To investigate the role of GRP78 in regulating cell proliferation under severely hypoxic conditions, GRP78(+/+) and GRP78(−/−) cells were cultured under severe hypoxia and treated with various concentrations of DDP for 24 h. As demonstrated by MTT assay (Fig. 3B), DDP inhibited the proliferation of GRP78(+/+) and GRP78(−/−) cells in a dose-dependent manner. However, the proliferation inhibition rates of GRP78(+/+) cells under severe hypoxia were markedly lower than those under normoxic conditions (P<0.01; Fig. 3A), indicating that severe hypoxia is a major cause of chemoresistance of hypopharyngeal carcinoma cells to DDP. Moreover, a stronger inhibitory effect was observed in GRP78(−/−) cells compared with that in GRP78(+/+) cells (P<0.01), indicating that induction of GRP78 under hypoxia impacts the chemosensitivity of hypopharyngeal carcinoma cells to DDP. Consistently, DDP induced apoptosis in GRP78(+/+) and GRP78(−/−) cells under normoxic and severely hypoxic conditions (Figs. 4A and B). However, the apoptosis rates induced by DDP were markedly less in GRP78(+/+) cells (P<0.01) compared with GRP78(−/−) cells under severe hypoxia. These observations indicate that GRP78 confers hypopharyngeal carcinoma cells to chemoresistance induced by severe hypoxia and knockdown of GRP78 sensitized DDP-induced apoptosis of hypopharyngeal carcinoma cells under severely hypoxic conditions.

### GRP78 regulates the expression of CHOP, Bcl-2 and Bax under severe hypoxia

**Figure 5:**
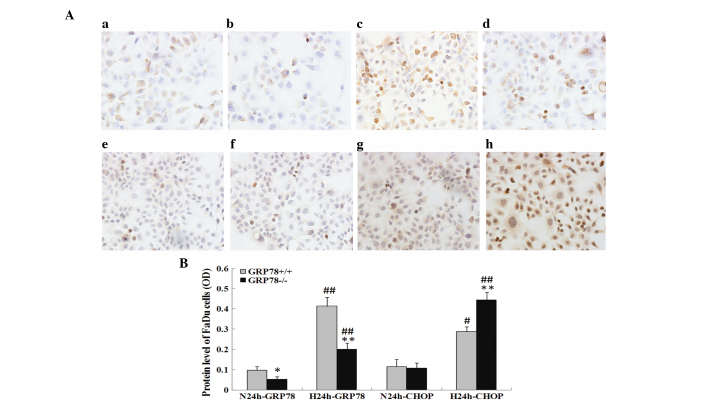
Immunohistochemical detection of GRP78 and CHOP protein expression levels in GRP78(+/+) and GRP78(−/−) hypopharyngeal carcinoma cells under 24 h of normoxic or severely hypoxic conditions. (A) Images were captured from representative immunohistochemical staining of GRP78 and CHOP proteins: (a) Positive expression of GRP78 under N24h in GRP78(+/+) cells; (b) low expression of GRP78 under N24h in GRP78(−/−) cells; (c) high expression of GRP78 under H24h in GRP78(+/+) cells; (d) low expression of GRP78 under H24h in GRP78(−/−) cells; (e) low expression of CHOP under N24h in GRP78(+/+) cells; (f) low expression of CHOP under N24h in GRP78(−/−) cells; (g) positive expression of CHOP under H24h; and (h) high expression of CHOP under H24h in GRP78(−/−) cells (magnification, ×200). (B) Histogram of the expression levels of GRP78 and CHOP proteins in GRP78(+/+) and GRP78(−/−) cells. ^*^P<0.05 and ^**^P<0.01, vs. GRP78(+/+); ^#^P<0.05 and ^##^P<0.01, vs. N24h. Data shown are from three individual experiments. GRP78, glucose-regulated protein 78; CHOP, C/EBP homology protein; OD, optical density; N24h, normoxia for 24 h; H24h, severe hypoxia for 24 h.

Expression of GRP78 and CHOP in GRP78(+/+) and GRP78(−/−) cells under severe hypoxia was initially investigated by immunohistochemistry. It was revealed that the expression of GRP78 was mainly located in the cytoplasm or cytomembrane, whereas the expression of CHOP was mainly found in the cytoplasm or nucleus (Fig. 5A). Compared with normoxic conditions, severe hypoxia induced a significant increase in the protein expression levels of GRP78 (P<0.01) and CHOP (P<0.05) (Fig. 5B). Notably, knockdown of GRP78 led to significantly increased CHOP expression under severe hypoxia (P<0.01; Figs. 5A and B).

**Figure 6:**
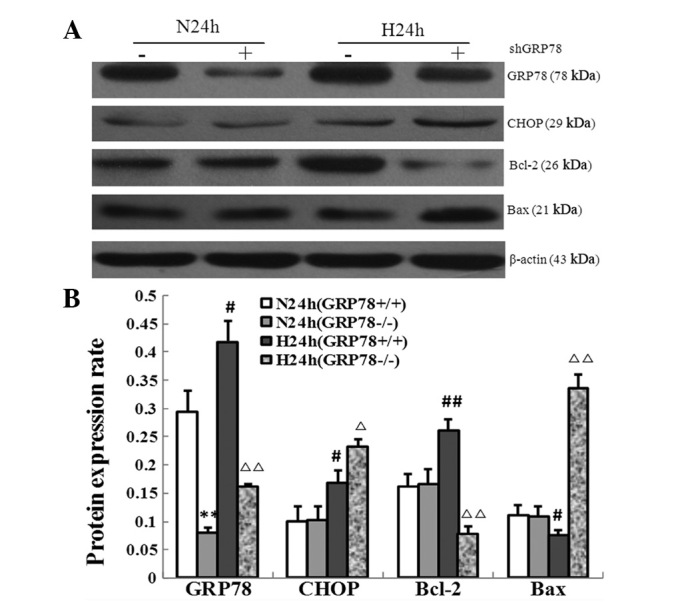
Changes in the expression of apoptosis-regulating proteins following the knockdown of GRP78 with shRNA. (A) Protein levels of GRP78, CHOP, Bcl-2 and Bax following knockdown of GRP78 in hypopharyngeal carcinoma cells were measured by immunoblot analysis under normoxic and severely hypoxic cultures for 24 h. (B) Histogram of the protein expression levels of GRP78, CHOP, Bcl-2 and Bax in GRP78(+/+) and GRP78(−/−) cells under normoxia and severe hypoxia for 24 h. ^**^P<0.01, vs. N24h GRP78(+/+); ^#^P<0.05 and ^##^P<0.01, vs. N24h GRP78(+/+); ^Δ^P<0.05 and ^ΔΔ^P<0.01, vs. H24h GRP78(+/+). Data shown are from three individual experiments. GRP78, glucose-regulated protein 78; CHOP, C/EBP homology protein; shRNA, short hairpin RNA; N24h, normoxia; H24h, severe hypoxia.

The expression of GRP78 and CHOP and their downstream effector proteins, Bcl-2 and Bax, in GRP78(+/+) and GRP78(−/−) cells was also investigated by western blot analysis following incubation under normoxic and severely hypoxic conditions (Figs. 6A and B). It was found that GRP78 expression was significantly decreased in GRP78(−/−) cells (P<0.01). The increase in the protein level of CHOP was observed under severe hypoxia in GRP78(+/+) cells (P<0.05), however, it was more prominent in GRP78(−/−) cells. This indicated that knockdown of GRP78 may promote the expression of CHOP in the later stages of UPR induced by hypoxia. Blocking GRP78 by shRNA also resulted in the downregulation of Bcl-2 and upregulation of Bax under severe hypoxia (all P<0.01).

## Discussion

HNSCC ranks as the sixth most common malignancy of the human body worldwide and exhibits formidable features of resistance to chemotherapeutic agents (11). Hypoxia is a major cause of therapeutic resistance and prevails in the tumor bulks simultaneously with the development and progression of solid tumors, including hypopharyngeal carcinoma. Typically, tumor cells suffer from stresses, including glucose starvation, hypoxia and oxidative stress, due to an insufficient supply of nutrients, which activates intracellular signaling pathways, including UPR, to overwhelm intra-plasma disorders and protect against cell death (12).

UPR is a biological behavior of cancer cells, which functions to degrade unfolded protein for the preservation of energy and activation of anti-apoptotic factors. For example, GRP78, the most important marker of UPR, has been identified as a survival factor for rescuing cells from cell death and overexpression of GRP78 has been reported to be associated with poor prognosis in a number of malignant tumors (13,14). Nevertheless, the role of GRP78 in regulating the proliferation and growth of hypopharyngeal carcinoma cells under severe hypoxia remains to be unveiled. The present study demonstrated that hypopharyngeal carcinoma cells are more resistant to DDP under severe hypoxia, which was made evident by results from the proliferation inhibition and apoptosis assays. In addition, the rates of proliferation inhibition and apoptosis induced by DDP were markedly increased in GRP78(−/−) cells, indicating that the induction and activation of GRP78 is responsible for chemoresistance in response to severe hypoxia.

Activation of GRP78 may vary with the severity of hypoxia (15). It has been observed in the present study that GRP78 was substantially upregulated under severe hypoxia when compared with normoxia, which is consistent with observations from previous studies (16). By contrast, normoxia or moderate hypoxia brought almost no change to GRP78 expression. Notably, it was found that hypoxic conditions (oxygen concentration, <0.02%) are necessary for UPR activation. Therefore, severe hypoxia rather than moderate hypoxia leads to UPR activation in hypopharyngeal carcinoma cells.

In addition, the present study revealed that severe hypoxia leads to the induction of CHOP, an additional significant regulating protein of the proapoptotic arm of UPR. Moreover, the induction of CHOP was preceded by the upregulation of GRP78, indicating that the gene expression of UPR in response to severe hypoxia is during dynamic changes, depending on the stage and status of hypoxia. This is partly reflected by the corresponding changes observed in HIF-1α, an important marker of hypoxia.

To further explore the mechanism of the knockdown of GRP78 for the sensitization of hypopharyngeal carcinoma cells to DDP under severe hypoxia, the interaction between GRP78 and CHOP and its effects on the downstream apoptosis-regulating proteins Bcl-2 and Bax was investigated. A previous study by Pyrko *et al* demonstrated that the inhibition of GRP78 may upregulate CHOP expression in the absence of any treatment under normoxic conditions in malignant glioma cells (17). Similarly, the current study found that the expression levels of GRP78 and CHOP were significantly increased under severe hypoxia and downregulation of GRP78 by shRNA was accompanied with the simultaneous upregulation of CHOP. The results indicate that similar regulating mechanisms may exist between GRP78 and CHOP in hypopharyngeal carcinoma cells and a checkpoint of the pro-apoptosis pathway is initiated when the pro-survival pathway, dominated by GRP78, is blocked under severe hypoxia.

Elevated GRP78 expression prevents tumor cells from stress-induced apoptosis, suppressing apoptosis and protecting cells against cisplatin and hypoxia. It has been previously reported that a fraction of GRP78 exists as an ER transmembrane protein that forms a complex with caspase-7 or caspase-12. The formation of this complex is dependent on its ATP binding domain and directly inhibits the activity of proapoptotic effectors localized in the ER (18). GRP78 overexpression inhibits Bax activation, which is also occurring in hypopharyngeal carcinoma cells under severe hypoxia as observed in the present study. In addition, knockdown of GRP78 leads to Bax activation (19) and provokes cytochrome *c* release from the mitochondria. Functionally, GRP78 and antiapoptotic protein Bcl-2, are capable of forming separate complexes with BIK. Elevated GRP78 expression leads to a reduction of Bcl-2 binding to BIK and Bcl-2 sequestration by BIK at the endoplasmic reticulum (20), which prevents endoplasmic reticulum Ca^2+^ release, thereby, suppressing apoptosis. Downregulation of GRP78 may upregulate the pro-apoptosis factor of CHOP, which has been reported to downregulate the expression of antiapoptotic Bcl-2 (21) and enhance apoptosis. In a previous study analyzing heterozygous knockout mice, it was identified that a lower level of GRP78 leads to the specific induction of CHOP and activation of executioner caspase-3 and caspase-7 (22). CHOP is also known as growth arrest and DNA damage-inducible gene 153, which implies that it induced apoptosis through direct inhibition, causing DNA damage. The crosstalk between the mitochondria and ER through interactions between GRP78 and CHOP determines the fate of the cell growth and proliferation of hypopharyngeal carcinoma cells following treatment with DDP in response to severe hypoxia.

In conclusion, induction of GRP78 through UPR is determined by the severity of hypoxia. Severe hypoxia causes UPR with induction of GRP78 expression in hypopharyngeal carcinoma, which is a major cause of chemoresistance to DDP. Downregulation of GRP78 upregulates CHOP and the proapoptotic factor Bax, inhibiting the expression of the antiapoptotic factor Bcl-2, thus, resulting in the inhibition of cell growth and proliferation. Knockdown of GRP78 sensitizes hypopharyngeal carcinoma cells to DDP treatment and alleviates hypoxia-induced chemoresistance to DDP. Therefore, downregulation of GRP78 may become a promising adjuvant treatment strategy for overcoming the therapeutic resistance of HNSCC.

## References

[1] Chien CY, Su CY, Hwang CF, Chuang HC, Hsiao YC, Wu SL, Huang CC (2006). Clinicopathologic significance of CD105 expression in squamous cell carcinoma of the hypopharynx. Head Neck.

[2] Brown JM, Wilson WR (2004). Exploiting tumour hypoxia in cancer treatment. Nat Rev Cancer.

[3] Wouters BG, Koritzinsky M (2008). Hypoxia signalling through mTOR and the unfolded protein response in cancer. Nat Rev Cancer.

[4] Ghosh R, Lipson KL, Sargent KE, Mercurio AM, Hunt JS, Ron D, Urano F (2010). Transcriptional regulation of VEGF-A by the unfolded protein response pathway. PLoS ONE.

[5] Rzymski T, Milani M, Pike L (2010). Regulation of autophagy by ATF4 in response to severe hypoxia. Oncogene.

[6] Koumenis C, Wouters BG (2006). ‘Translating’ tumor hypoxia: unfolded protein response (UPR)-dependent and UPR-independent pathways. Mol Cancer Res.

[7] Rutkowski DT, Kaufman RJ (2004). A trip to the ER: coping with stress. Trends Cell Bio.

[8] Grkovic S, O’Reilly VC, Han S, Hong M, Baxter RC, Firth SM (2012). IGFBP-3 binds GRP78, stimulates autophagy and promotes the survival of breast cancer cells exposed to adverse microenvironments. Oncogene.

[9] Kandala PK, Srivastava SK (2012). Regulation of macroautophagy in ovarian cancer cells in vitro and in vivo by controlling glucose regulatory protein 78 and AMPK. Oncotarget.

[10] de Ridder G, Ray R, Misra UK, Pizzo SV (2011). Modulation of the unfolded protein response by GRP78 in prostate cancer. Methods Enzymol.

[11] Jemal A, Siegel R, Ward E, Hao Y, Xu J, Murray T, Thun MJ (2008). Cancer statistics, 2008. CA Cancer J Clin.

[12] Sun Q, Li X, Lu X, Di B (2011). Cancer stem cells may be mostly maintained by fluctuating hypoxia. Med Hypotheses.

[13] Huang LW, Lin CY, Lee CC, Liu TZ, Jeng CJ (2012). Overexpression of GRP78 is associated with malignant transformation in epithelial ovarian tumors. Appl Immunohistochem Mol Morphol.

[14] Kuroda K, Horiguchi A, Asano T (2011). Glucose-regulated protein 78 positivity as a predictor of poor survival in patients with renal cell carcinoma. Urol Int.

[15] Koumenis C, Bi M, Ye J, Feldman D, Koong AC (2007). Hypoxia and the unfolded protein response. Methods Enzymol.

[16] Krivoruchko A, Storey KB (2013). Activation of the unfolded protein response during anoxia exposure in the turtle *Trachemys scripta elegans*. Mol Cell Biochem.

[17] Pyrko P, Schönthal AH, Hofman FM, Chen TC, Lee AS (2007). The unfolded protein response regulator GRP78/BiP as a novel target for increasing chemosensitivity in malignant gliomas. Cancer Res.

[18] Healy SJ, Gorman AM, Mousavi-Shafaei P, Gupta S, Samali A (2009). Targeting the endoplasmic reticulum-stress response as an anticancer strategy. Eur J Pharmacol.

[19] Fu Y, Li J, Lee AS (2007). GRP78/BiP inhibits endoplasmic reticulum BIK and protects human breast cancer cells against estrogen starvation-induced apoptosis. Cancer Res.

[20] Zhou H, Zhang Y, Fu Y, Chan L, Lee AS (2011). Novel mechanism of anti-apoptotic function of 78-kDa glucose-regulated protein (GRP78): endocrine resistance factor in breast cancer, through release of B-cell lymphoma 2 (BCL-2) from BCL-2-interacting killer (BIK). J Biol Chem.

[21] McCullough KD, Martindale JL, Klotz LO, Aw TY, Holbrook NJ (2001). Gadd153 sensitizes cells to endoplasmic reticulum stress by down-regulating Bcl2 and perturbing the cellular redox state. Mol Cell Biol.

[22] Dong D, Ni M, Li J (2008). Critical role of the stress chaperone GRP78/BiP in tumor proliferation, survival, and tumor angiogenesis in transgene-induced mammary tumor development. Cancer Res.

